# Role of Conventional Dynamic Myelography for Detection of High-Flow Cerebrospinal Fluid Leaks: Optimizing the Technique

**DOI:** 10.1007/s00062-020-00943-w

**Published:** 2020-08-26

**Authors:** Eike I. Piechowiak, Katarzyna Pospieszny, Levin Haeni, Christopher M. Jesse, Giovanni Peschi, Pascal J. Mosimann, Johannes Kaesmacher, Pasquale Mordasini, Andreas Raabe, Christian T. Ulrich, Jürgen Beck, Jan Gralla, Tomas Dobrocky

**Affiliations:** 1 University Institute of Diagnostic and Interventional Neuroradiology, Inselspital, Bern University Hospital, University of Bern, Freiburgstrasse 8, 3010 Bern, Switzerland; 2 Department of Neurosurgery, Inselspital, Bern University Hospital, University of Bern, Bern, Switzerland; 3 Department of Interventional, Pediatric and Diagnostic Radiology, Inselspital, University Hospital, University of Bern, Bern, Switzerland; 4 Department of Neurosurgery, Medical Center—University of Freiburg, Freiburg, Germany

**Keywords:** Spontaneous intracranial hypotension, CSF leak, Conventional dynamic myelography

## Abstract

**Background:**

Spinal imaging is essential to identify and localize cerebrospinal fluid (CSF) leaks in spontaneous intracranial hypotension (SIH) patients when targeted treatment is necessary.

**Purpose:**

Provide an in-depth presentation of the conventional dynamic myelography (CDM) technique for localizing spinal CSF leaks in SIH patients.

**Material and Methods:**

Consecutive SIH patients with a CSF leak confirmed on CDM and postmyelography computed tomography (CT) investigated at our institution between 2013 and 2019 were retrospectively analyzed. Intraoperative reports were reviewed to confirm the accuracy of CDM.

**Results:**

In total, 62 patients (mean age 45 years) were included; 48 with a ventral dural tear, 12 with a meningeal diverticulum, and in 2 patients positive for spinal longitudinal extradural CSF collection the site remained unclear. The leak was identified during the first and the second CDM in 43 and 17 patients, respectively. The use of CDM correctly identified the site of the CSF leak in all but one patient undergoing surgical closure (45/46, 98%). The mean fluoroscopy time was 7.8 min (range 1.8–14.4 min) with a radiation dose for a single examination of 310 mGy (range 28–1237 mGy).

**Conclusion:**

The CDM procedure has a high accuracy for spinal CSF leak localization including dural tears and spinal nerve diverticula. It is the technique with the highest temporal resolution, is robust to breathing artifacts, allows great flexibility regarding patient positioning, compares favorably to other dynamic examinations with respect to the radiation dose and does not require general anesthesia. For CSF venous fistulas, however, other dynamic examinations, such as digital subtraction myelography, seem more appropriate.

**Video online:**

The online version of this article contains 4 videos. The article and the videos are online available (10.1007/s00062-020-00943-w). The videos can be found in the article back matter as “Electronic Supplementary Material”.

## Introduction

Spontaneous intracranial hypotension (SIH) is a well-recognized disorder usually presenting with disabling orthostatic headache that manifests within minutes after assuming the upright position and subsides after lying down. Schievink et al. proposed three types of spontaneous spinal CSF leaks: type 1) the dural tear, type 2) the meningeal diverticulum and type 3) the CSF-venous fistula [1]. When conservative measures, such as bed rest and caffeine do not provide long-term symptom relief, epidural blood patches or definitive microsurgical closure of the dural tear may be necessary. In these cases, precise localization of the leakage site is necessary. Unenhanced spine magnetic resonance imaging (MRI) is an excellent non-invasive method, which enables identification of epidural CSF collection, but lacks temporal resolution [2]. Intrathecal gadolinium-enhanced myelography (GdM) and postmyelography computed tomography (PMCT) provide spatial resolution allowing visualization of epidural contrast accumulation; however, only a few minutes of delay between intrathecal contrast application and imaging may obscure the leakage point, since contrast may span several vertebral levels, making localization impossible.

To overcome this difficulty methods with high temporal resolution, which can pinpoint the level of spinal dural breach, such as conventional dynamic myelography (CDM) [3], digital subtraction myelography (DSM) [4–6], and dynamic computed tomography myelography (DCTM) [7–9] have been reported. The DSM has a high temporal resolution but is susceptible to breathing and motion artifacts. Although DCTM is a valuable adjunctive technique, which combines high temporal and spatial resolution, it is associated with a higher radiation dose [7]. Thus, DCTM should cover only a few vertebral levels suspected of harboring discogenic microspurs or perineural cysts. Detailed descriptions of the CDM technique in a large patient cohort with SIH is lacking, and its use varies between institutes.

The goal of our study was therefore to review the role of CDM in SIH patients and provide a detailed description of the application of this technique to confirm and localize a spinal CSF leak.

## Material and Methods

This study was granted institutional review board approval, and the need for informed consent was waived owing to its retrospective nature. The registry was approved by the local ethics committee.

All consecutive SIH patients investigated at our institution between February 2013 and January 2019 with positive spinal imaging (CSF leak identified on CDM and PMCT) were retrospectively reviewed. No additional exclusion criteria were applied.

Subgroups of patients have previously been included in other studies, which have investigated different outcome measures, including optic nerve sheath ultrasonography, surgical details of dural closure, CSF dynamics, and brain MRI, spine MRI, but which did not report on the imaging aspects of CDM in particular [2, 7, 10–15].

### Diagnostic Work-up

A detailed medical history was obtained, then physical examination, optic nerve sheath ultrasonography, and lumbar infusion testing were performed [10, 11]. For most patients, a brain MRI was acquired to rule out any underlying intracranial pathology and the SIH score was calculated. The SIH score is based on the 6 most relevant imaging findings; 3 major (2 points each): pachymeningeal enhancement, engorgement of venous sinus, and effacement of the suprasellar cistern (≤4.0 mm), and 3 minor (1 point each): subdural fluid collection, effacement of the prepontine cistern (≤5.0 mm), and mamillopontine distance (≤6.5 mm). The SIH score helps to predict the likelihood of a CSF leak [15]. Spinal imaging began with an unenhanced MRI including three sagittal T2-weighted spin echo (SE) blocks and an isotropic 3D T2-weighted turbo spin echo sequence with fat saturation covering the brain and entire spine. Unenhanced spine MRI was routinely performed in the morning and GdM in the afternoon of the same day after intrathecal injection of a mixture of 0.5ml Gadovist (gadobutrol, 1.0 mmol/ml, Bayer, Leverkusen, Germany—nonionic macrocyclic agent) or 0.5 ml Magnograf® (gadopentetate dimeglumine, 0.5 mmol/ml, Berlis AG, Zurich, Switzerland) mixed with 9.5 ml of CSF.

The CDM was performed on a monoplane high-resolution angiographic system (Artis zee multipurpose; Siemens, Erlangen, Germany) equipped with a flat panel detector (30 × 40 cm) by two board certified neuroradiologists (E.I.P and T.D with 10 and 8 years of experience, respectively). To facilitate orientation during CDM and postinterventional image analysis, a small radiopaque skin marker was taped to the patient’s back just off the midsagittal plane, at the level of the most suspicious lesion (e.g. microspur, nerve root sleeve diverticulum), considered by the operator as potentially responsible for the leak.

**Fig. 1:**
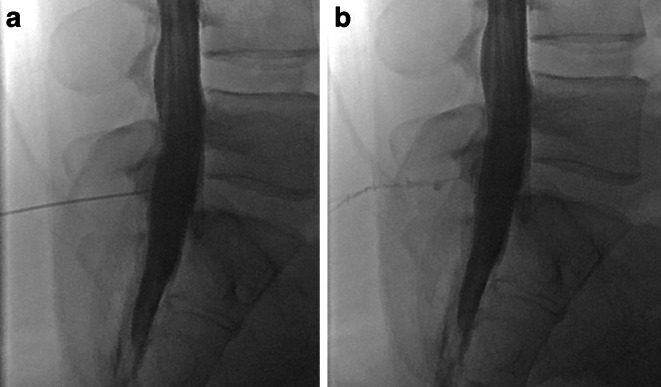
Female with known migraine. **a** Intrathecal injection of the contrast medium in sitting position at the level L5/S1 with the puncture needle still in place. **b** Contrast medium leakage after retraction of the needle along the puncture channel into the posterior paravertebral soft tissue and the epidural space

Lumbar puncture was performed with the patient in a sitting position and, after intrathecal injection of 20 ml Iopamiro 300 (Iopamidol, Bracco, Switzerland), the needle was withdrawn. Since its specific gravity is higher than that of CSF, layering of contrast material in the dependent portion of the thecal sac occurs. To prevent false positive results due to contrast leakage at the level of the lumbar puncture, which may occur during needle withdrawal (Fig. 1 + case video 1), an atraumatic 25G spinal needle (Pencan, B. Braun, Melsungen, Germany) or 22G spinal needle (Ago spinale, PIC solution, Grandate, Italy) were routinely used. In addition, after intrathecal administration of iodinated contrast medium, the patient was asked to perform a Valsalva maneuver in a sitting position, while the operator looked for contrast leakage into the epidural space at the lumbar level. If no leakage was observed on fluoroscopy, and PMCT demonstrated extrathecal contrast confined to the level of the puncture site and the adjacent 1–2 vertebral levels, it was considered iatrogenic.

**Fig. 2:**
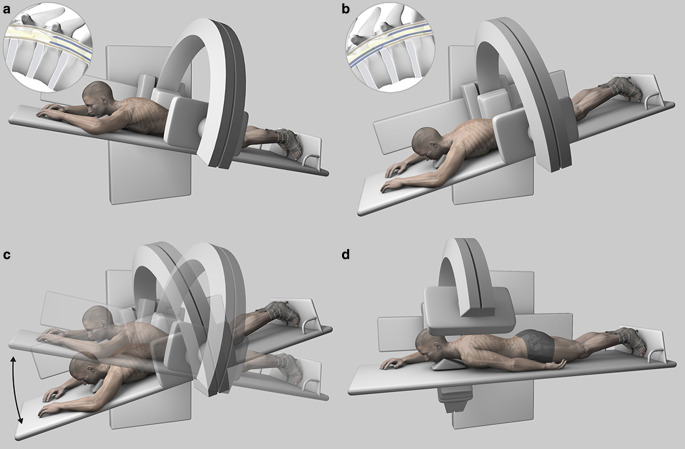
A patient with a suspected ventral spinal CSF leak, in the prone decubitus position. **a** The patient’s feet are secured to the table using adjustable straps and both arms are extended above the head. The head is tilted upwards and supported with a foam wedge to prevent excessive flow of contrast material into the intracranial subarachnoid space. Lateral projection is used. The inlay in the upper left corner shows intradural contrast media (in blue) outlining the myelon. **b** The table is progressively tilted into the Trendelenburg position. The inlay in the upper left corner shows leakage of contrast medium into the ventral epidural space. **c** Illustration of the progressive tilting of the table and cranial movement of the detector following the leading edge of the contrast material. **d** For better image quality at the cervicothoracic junction, the left arm is placed alongside the body and the X‑ray tube is slightly rotated to a right oblique view

**Fig. 3:**
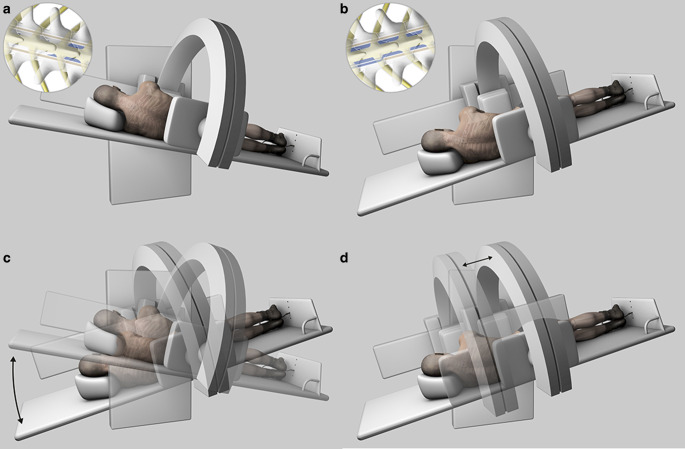
A patient with suspected leakage from the nerve root sleeve diverticulum, in the lateral decubitus position. **a** The patient’s feet are secured to the table using adjustable straps and both arms are positioned in front of the chest. The head is tilted upwards and supported with a foam wedge to prevent excessive flow of contrast material into the intracranial subarachnoid space. The inlay in the upper left corner shows intradural contrast media (in blue) outlining the myelon. **b** The table is progressively tilted into the Trendelenburg position. The inlay in the upper left corner shows leakage of contrast media into the lateral epidural space. **c** Illustration of the progressive tilting of the table and cranial movement of the detector following the leading edge of the contrast material. **d** Illustration showing the cranial movement of the detector following the leading edge of the contrast medium

**Fig. 4:**
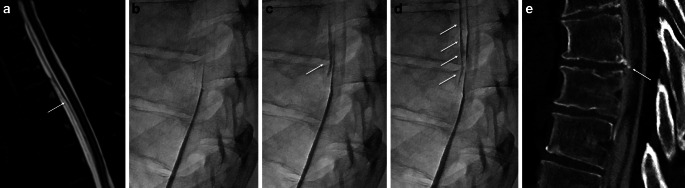
Middle-aged patient with sudden onset of orthostatic headache. **a** Heavily T2 weighted sagittal MRI with visible cerebrospinal fluid in the ventral epidural space, and the dura mater (*arrow*). **b**–**d** Dynamic myelography with contrast medium leaking into the ventral epidural space (*arrow*). **e** Postmyelography CT demonstrating a ventral microspur (*arrow*) at the corresponding level leading to a dural breach

**Fig. 5:**
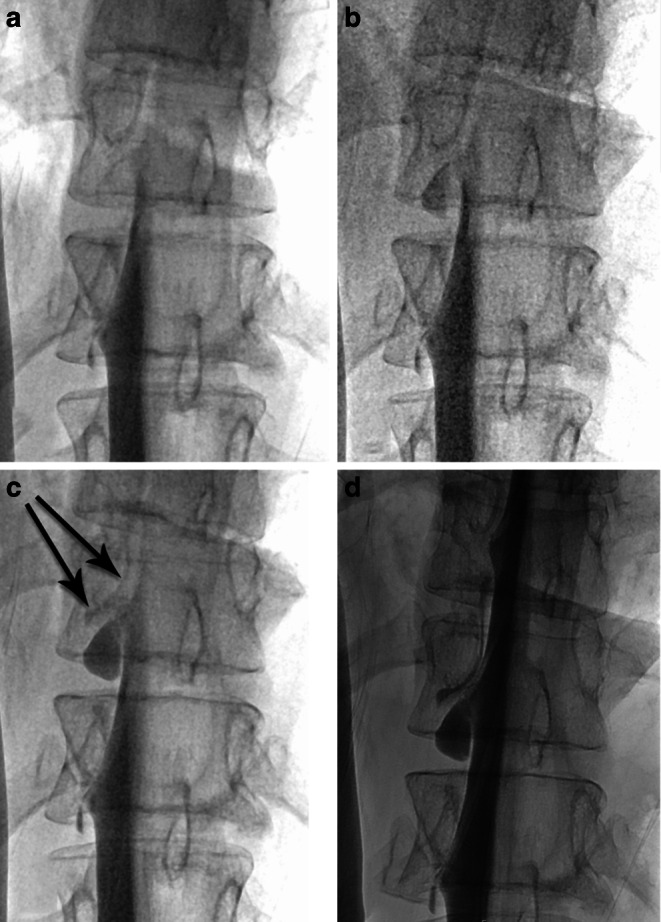
Middle-aged female with occipital headache and vertigo she had been suffering for 1 year. Dynamic myelography in right decubitus with progressive tilt of the table with time course from **a**–**d**, total time: 14 s. **b** Filling of the nerve root diverticulum with contrast medium. **c** Contrast medium leaking into the epidural space adjacent to the diverticulum with progressive extension more cranially (*black arrows*). **d** Final image demonstrating contrast media distribution in the epidural space

For the dynamic part of the investigation, patient positioning was adapted according to the findings of the previous spinal MRI; prone when a ventral osteodiscogenic microspur was suspected, or lateral decubitus when a ruptured spinal nerve root diverticulum was the presumed source of the leakage. In both positions, the head was tilted upwards and supported with a foam wedge to prevent excessive flow of contrast agent into the intracranial subarachnoid space. Lateral projection was used in patients in the prone position and anteroposterior projection in patients in lateral decubitus, which provided optimal conditions for identifying the leakage point when contrast spillage occurs perpendicularly to the incidental X‑ray beam. The patient’s feet were secured to the table using adjustable straps to prevent sliding (Foot Holder, 74 29 830, Siemens Healthcare, Erlangen, Germany) and both arms were extended above the head. The table was progressively tilted into the Trendelenburg position (approximately 30–45°) to facilitate intrathecal caudocranial flow of the injected contrast medium (Fig. 2 and 3). During tilting, pulsed fluoroscopy was performed with optimized collimation. The default frame rate was 7.5 f/s, and the detector was slowly moved in a cranial direction following the leading edge of the contrast medium along the ventral or lateral margin of the thecal sac. Fluoroscopic sequences were intermittently stored. The level at which contrast material first exited the intrathecal compartment and started filling the epidural space was defined as the level of leakage. As soon as spillage occurred one single-shot image was acquired. (ventral microspur Fig. 4 and case video 2; leaking spinal nerve root diverticulum Fig. 5 and case video 3). When the leakage point was not identified during the first run, the table was tilted back to the reverse Trendelenburg position for 3 min to allow gravity-dependent layering of contrast medium in the dependent portion of the thecal sac. Before performing a second run and tilting the table back to the Trendelenburg position, patient positioning, collimation and zoom were adjusted.

For better image quality at the cervicothoracic junction, which may be reduced due to superimposed bony structures of the shoulders and rib cage, the left arm was placed alongside the body and the X‑ray tube was slightly rotated to an oblique orientation (Fig. 2d).

**Fig. 6:**
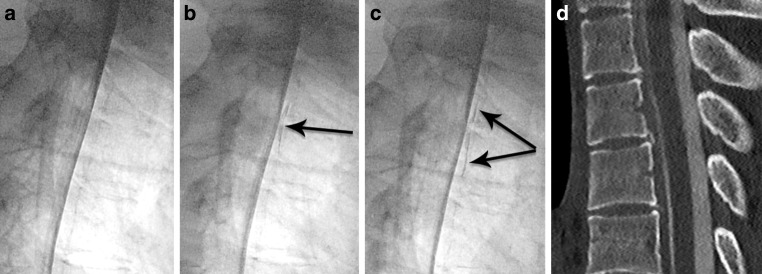
Middle-aged female with sudden onset of orthostatic headache after a mild trauma. Dynamic myelography in prone position with progressive tilt of the table with time course from **a**–**c**, total time: 10 s. **a** Before and **b** immediately after Valsalva maneuver (*black arrow* showing the precise location of the leak) with increasing leakage of contrast medium (*black arrows*) into the ventral epidural space **c**. **d** Postmyelography CT demonstrating a prominent ventral microspur at the corresponding level

In selected patients in whom an intermittent leak with a valve mechanism (e.g. ventral cord hernia) was suspected, a Valsalva maneuver was performed [16]. (Fig. 6 and case video 4).

The patient was immediately transferred to the CT imaging suite and PMCT, usually with the patient in the decubitus position, was performed on a 128-multidetector CT (SOMATOM Definition Edge, Siemens, Erlangen, Germany) to identify possible causative pathology at the level of dural dehiscence. If no epidural contrast was identified in the first PMCT, a late phase PMCT was performed in general 4–12 h after initial intrathecal injection to exclude low flow leaks.

### Repeat Examinations

If epidural contrast accumulation was demonstrated on PMCT, but after review of all fluoroscopic sequences the precise site of the leak remained undetermined, the procedure was repeated if the performing neuroradiologist considered that a specific area could be better assessed on a second run. The re-examination was usually performed on the following day (>24 h) to allow for resorption of intrathecal contrast material. The technique was adjusted according to the results of the previous examination (i.e., patient positioning, higher zoom, X‑ray tube angulation, focusing on a smaller area). If the neuroradiologist believed that owing to overprojection of bony structures usually present at the cervicothoracic junction this specific area should be reassessed, a DCTM was performed as DSM was not part of our routine diagnostic work-up during the study period.

### Standard of Reference

For all patients with a confirmed leak on CDM/PMCT undergoing microsurgical closure of the dural dehiscence, intraoperative reports were reviewed. This was done to confirm the presence of epidural CSF and verify the level of dural dehiscence, since operative exploration remains the gold standard. In patients managed conservatively or with epidural blood patching, two neuroradiologists (E.I.P and T.D) established the standard of reference through the combination of multimodal imaging (unenhanced MRI, GdM, CDM, PMCT), since the sensitivity of each individual method is not clear. When a disagreement between different imaging modalities was identified, the finding was reviewed together to reach a consensus.

### Data Analysis

All imaging studies were reviewed independently on a picture archiving and communication system (PACS) station (R11.4.1, 2009; Philips, Best, Netherlands; Sectra, Linkoping, Sweden) by two board-certified neuroradiologists (E.I.P and K.P). Precise location of the CSF leak as demonstrated on CDM, and the underlying pathology as seen on PMCT, were recorded. Conflicts were resolved by consensus. Demographic data including patient age, and sex were recorded. Fluoroscopy-specific data reported included patient positioning, number of CDM examinations, and periprocedural complications. Patient radiation dose was approximated by indirect measures of radiation output from the angiographic system including fluoroscopy time (FT), cumulative air kerma (CAK in mGy), and kerma area product (KAP in μGy/m^2^). Intraoperative reports were reviewed to confirm the presence of epidural CSF and verify the level of dural dehiscence. Descriptive statistics were calculated using Microsoft Excel 2016. Continuous variables were described as a mean ± standard deviation (SD) and categorical variables as frequencies (percentage).

## Results

A total of 62 SIH patients with a spinal CSF leak confirmed on CDM and PMCT were included, 44 female and 18 male. The mean patient age was 47 years (range 26–73 years, female 47 years, range 26–73 years, male 47 years, range 26–61 years). The mean brain SIH score was 6.6 (SD ± 2.23), indicating a high probability of a spinal CSF leak. The mean number of CDM examinations per patient was 2 (range 1–4) with a mean FT of 7.8 min (range 1.8–14.4 min). The radiation dose for a single examination was 310 mGy (range 28–1237 mGy). The leak was identified during the first CDM examination in 43, and during the second CDM in 17 patients.

The site of CSF leakage was at the cervical, thoracic, and lumbar levels in 4, 49, and 7 patients, respectively. In two patients positive for spinal longitudinal extradural CSF collection on PMCT, the level of leakage remained unclear. These were investigated at the beginning of the study period and were not repeated due to symptom improvement. A ventral dural tear was identified in 48 patients (46 ventral tear due to a calcified discogenic microspur) and a leaking meningeal diverticulum in 12 patients. In 45 out of 46 patients undergoing microsurgical closure, CDM correctly identified the leakage site, in 1 patient the leakage site was one level off. One patient who underwent two CDM examinations on the following day (>24 h) had transient aphasia after the second examination, most likely an adverse reaction to intrathecal contrast medium administration: the brain MRI did not reveal any suspicious findings. No other serious periprocedural complications were reported.

In 19 other patients not included in this study, in whom CDM was insufficient for precise localization of the CSF leak either due to superimposed bony structures of the shoulder and rib cage or low-flow leaks, dynamic CT myelography was performed for leak localization and have been previously published [7].

### Case Examples

#### Patient 1: Classical Ventral Epidural CSF Leak Caused by a Microspur

A 54-year-old female patient presented with orthostatic headache that she had suffered for 20 weeks. Sagittal T2-weighted fat-suppressed spine MRI demonstrated a CSF collection in the epidural space spanning several levels (T1–T10). The CDM was performed with the patient in the prone position, focusing on these levels. During progressive table tilting, immediate contrast spillage into the ventral epidural compartment at the level of T7/8 occurred, confirming a type 1 leak according to Schievink et al. ([1]; Fig. 4, case video 2).

#### Patient 2: Leaking Spinal Nerve Root Diverticulum

Sagittal T2-weighted SE sequence of the thoracic spine demonstrated CSF in the epidural compartment. Coronal MRI T2-weighted sequence with fat saturation and curved maximum intensity projection additionally showed multiple prominent nerve root sleeve cysts. The CDM was performed in the left lateral decubitus position and demonstrated a large T9/10 diverticulum with contrast agent leakage into the epidural space and around the T9 pedicle (Fig. 5, case video 3).

## Discussion

In patients with intractable SIH that do not respond to conservative measures or to non-targeted epidural blood patching, microsurgical exploration and closure of the dural breach is the treatment of choice. To tailor the surgical approach, limit the extent of bone removal during surgery and the need for multilevel intraoperative exploration, pinpointing the site of dural dehiscence using dynamic imaging with high temporal resolution is necessary [17]. This task can be challenging because communication between the intrathecal and epidural compartment may occur anywhere along the dural axis, although the vast majority are reported in the thoracic spine and cervicothoracic junction [18]. The CDM, PMCT and DCTM are imaging modalities with high temporal resolution and have been used for spinal CSF leak detection. Each modality has its unique strengths and shortcomings, should be considered.

The DSM has a limited area of coverage inherent to the size of the flat panel detector. In contrast, when using CDM the detector may be moved following the leading edge of the contrast media flowing in the cranial direction, thus allowing the entire length of the spine to be covered. Second, the digital subtraction technique is sensitive to breathing and motion artifacts, which decrease image quality. Thus, DSM requires excellent patient cooperation and suspension of respiration. Some centers perform DSM with the patient under general anesthesia, since misregistration artifacts may conceal the site of leakage. Third, as previously reported in the neurointerventional literature, DSA is associated with significant radiation exposure [19, 20]. Maus et al. have shown that during transforaminal epidural steroid injections, DSA delivered approximately 2–4-fold more radiation than conventional fluoroscopy during a 5s exposure [21]. A limitation inherent to both CDM and DSM is the use of planar rather than cross-sectional imaging.

The DCTM is a valuable technique that combines high temporal and spatial resolution, and is used particularly at the cervicothoracic junction where superimposed bony structures of the shoulder and rib cage can obscure the leakage point on planar imaging [7]; however, owing to the time delay between scans it does not match the temporal resolution of real-time CDM where multiple frames are acquired per second. Furthermore, DCTM is associated with a higher radiation dose and should therefore cover only the few vertebral levels suspected of harboring the leakage site. In our clinical practice, DCTM is cautiously used in patients in whom previous methods have failed to identify the leakage point.

In our experience, in dural leaks (type 1) or meningeal diverticula (type 2), contrast leakage into the epidural space occurs almost instantaneously, spanning several vertebral levels within a few seconds [1]. In this respect, a major advantage of fluoroscopy is that it provides the highest temporal resolution with real-time demonstration of contrast medium flow and thus CDM is the primary dynamic tool when a type 1 or 2 CSF leak is suspected. When the site of epidural spillage is missed on the first examination, the level of the suspected leakage site can be narrowed down to a smaller area, which will be the focus during the repeat examination.

Concerning other underlying mechanisms of SIH, as reported by Kranz et al. CSF-venous fistulas (type 3) can be detected with both CTM and dynamic myelography [3]. Recently, Schievink et al. have reported lateral decubitus digital subtraction myelography to have a higher diagnostic yield to identify spinal CSF-venous fistulas [22]; however, in cases of CSF-venous fistulas, there is no epidural CSF distribution and these patients were therefore not included in our investigation and these cases are not the primary indication for CDM.

**Fig. 7:**
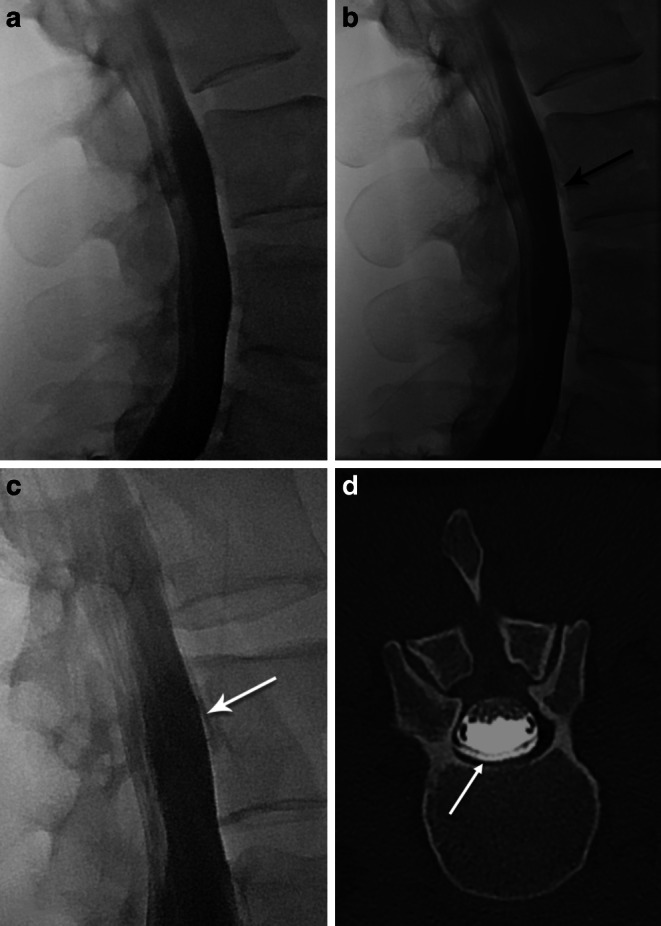
Middle-aged female with headache, neck pain, vertigo and nausea after spinal anesthesia. **a**–**c** Time course of dynamic myelography in sitting position with slow progression of contrast medium in the epidural space beginning at the level of vertebral body L3 **b** and progressing cranially **c** in the later phase. **d** Transversal postmyelography CT demonstrating contrast medium in the epidural space

When reporting PMCT and GdM, knowledge about the level of the lumbar puncture is essential to prevent false positive results, which may lead to confusion and unnecessary follow-up examinations. Extrathecal contrast in the epidural space confined to the level of the puncture site and the adjacent 1–2 vertebral levels should be considered iatrogenic. In patients referred for persisting post-dural puncture headache (e.g. after epidural anesthesia or spinal tap), lumbar puncture should be performed below the level of the previous puncture in order to distinguish between them (Fig. 7).

The major strength of our study is a meticulous description of the CDM technique, including useful illustrations of the workflow in patients with epidural CSF collection due to a dural breach. In addition, typical case examples are presented, which are accompanied by images and videos. In the majority of patients the leakage site was confirmed by microsurgical exploration, which underlines the accuracy of CDM. The limitation of this technique is the fact that is not reliable to demonstrate CSF-venous fistulas (type 3) as well as the retrospective and single center design which are inherent to the study design.

## Conclusion

The conventional dynamic myelography provides a real-time demonstration of contrast flow necessary to identify dural leaks and leaking meningeal diverticula, is robust to breathing artifacts, and does not require general anesthesia. When performed meticulously it is a valuable technique with a high accuracy for prospective identification of the site of cerebrospinal fluid leakage, and the radiation dose is lower than for digital subtraction myelography and dynamic CT myelography.

## Caption Electronic Supplementary Material

*Video 1*: middle-aged female with known migraine (same as in Fig. 1). Video demonstrating contrast medium leakage during retraction of the needle along the puncture channel into the paravertebral soft tissue and the epidural space

*Video 2*: middle-aged patient with sudden onset of orthostatic headache (same as in Fig. 4). Video sequence from dynamic myelography in prone position with progressive tilt of the table, demonstrating contrast medium leaking into the ventral epidural space

*Video 3*: middle-aged female with occipital headache and vertigo she has been suffering for 1 year (same as in Fig. 5). Dynamic myelography in right decubitus with progressive tilt of the table, demonstrating progressive filling of the nerve root diverticulum with contrast medium, and subsequent leakage into the epidural space adjacent to the diverticulum

*Video 4*: middle-aged female with sudden onset of orthostatic headache after a mild trauma (same as in Fig. 6). The initial dynamic myelography in prone position was unremarkable (not shown). The second run demonstrated an epidural leakage during the Valsalva maneuver

## Abbreviations

CAK: Cumulative air kerma
CDM: Conventional dynamic myelography
CSF: Cerebrospinal fluid
CT: Computed tomography
DCTM: Dynamic computed tomography myelography
FT: Fluoroscopy time
GdM: Intrathecal gadolinium enhanced myelography
KAP: Kerma area product
MRI: Magnetic resonance imaging
PACS: Picture archiving and communication system
PMCT: Postmyelography computed tomography
SE: Spin echo
SIH: Spontaneous intracranial hypotension
